# The Research Progress of Single-Molecule Sequencing and Its Significance in Nucleic Acid Metrology

**DOI:** 10.3390/bios15010004

**Published:** 2024-12-25

**Authors:** Yajun Wang, Jingjing Liu, Zhendong Wang, Mei Zhang, Yongzhuo Zhang

**Affiliations:** 1 College of Information and Control Engineering, Jilin Institute of Chemical Technology, Jilin 132022, China; wangyajun0813@163.com; 2 College of Information and Control Engineering, China Northeast Electric Power University, Jilin 132011, China; jingjing_liu@neepu.edu.cn; 3 Center for Advanced Measurement Science, National Institute of Metrology, Beijing 100029, China; 232086001021@lut.edu.cn (Z.W.); 18235289005@163.com (M.Z.)

**Keywords:** single-molecule sequencing, nucleic acid metrological traceability, nanopore

## Abstract

Single-molecule sequencing technology, a novel method for gene sequencing, utilizes nano-sized materials to detect electrical and fluorescent signals. Compared to traditional Sanger sequencing and next-generation sequencing technologies, it offers significant advantages, including ultra-long read lengths, rapid sequencing, and the absence of amplification steps, making it widely applicable across various fields. By examining the development and components of single-molecule sequencing technology, it becomes clear that its unique characteristics provide new opportunities for advancing metrological traceability. Notably, its direct detection capabilities offer a novel approach to nucleic acid metrology. This paper provides a detailed overview of library construction, signal generation and detection, and data analysis methods in single-molecule sequencing and discusses its implications for nucleic acid metrology.

## 1. Introduction

Gene sequencing technology began in the 1970s with the development of the first-generation Sanger sequencing method, based on the dideoxy technique [1]. While it offered high accuracy and specificity, it had limitations such as low throughput and short read lengths, making it unsuitable for processing large numbers of samples simultaneously. Second-generation sequencing technologies improved throughput through synthesis and sequencing techniques, becoming widely used in areas such as genetic disease diagnosis, infectious disease detection, cancer research, forensic medicine, and plant genetics [2,3,4]. However, second-generation sequencing still faced challenges, including read lengths of less than 500 bp, leading to errors from base insertions, deletions, and GC bias caused by PCR amplification. With advances in micro- and nano-processing and signal processing technologies, third-generation sequencing, based on single-molecule reading, emerged, overcoming the read length limitation and enabling ultra-long reads.

Advancements in science and technology have made life and health a global priority, with biometric traceability serving as a crucial foundation for ensuring the accuracy and reliability of clinical medical practices [5]. It plays a pivotal role not only in the prevention, diagnosis, and treatment of diseases but also in safeguarding public health. The International System of Units (SI)—encompassing meters, kilograms, seconds, amperes, kelvins, moles, and candelas—has entered the quantum era. This shift will streamline the quantum value transmission traceability process, making it faster, shorter, and more stable. Metrological traceability refers to the process of linking measurement results to reference objects through an uninterrupted calibration chain, as defined in the documentation. In a similar vein, in the field of life sciences, the goal of nucleic acid measurement traceability is to achieve the link between the nucleic acid quantity and the international basic unit, to ensure the accuracy, reliability and equivalent consistency of each nucleic acid detection result, and how to establish sequence traceability, which will have a profound impact on the biological industry and people’s life and health [6]. Single-molecule sequencing technology offers a novel approach to directly capture information from nucleic acid sequence variations without the need for sample amplification, thus paving the way for enhanced quantitative traceability.

Single-molecule sequencing, with its direct measurement of individual molecules and amplification-free process, simplifies the detection chain and reduces uncertainty introduced by amplification and other issues. As a rapidly advancing technology, single-molecule sequencing has shown various technical forms, each contributing to the future development of sequencing. Thanks to its unique nanostructure and high sensitivity, single-molecule sequencing has found applications across many fields, including materials science, energy, biomedicine, ecology, and security [7,8,9,10]. Due to its high precision and ability to obtain direct sample information, single-molecule sequencing provides a novel approach to nucleic acid sequence measurement, offering new possibilities for quantitative traceability.

## 2. Research Progress of Single-Molecule Sequencing Technology

Single-molecule sequencing technology refers to the method that enables the sequencing of individual nucleic acid molecules without the need for PCR amplification. This technology is also known as third-generation sequencing technology, which can theoretically determine the infinite length of a nucleic acid sequence. Unlike second-generation sequencing, which relies on fluorescence-based signal detection, single-molecule sequencing can be broadly categorized into two types based on different principles and materials: one utilizing fluorescence signal detection and the other employing electrical signal detection. These differing detection methods and technical principles impart unique characteristics to the entire sequencing process, from sample preparation to data acquisition.

Similarly, single-molecule sequencing technology, with its unique advantages, ensures that mutations are not artificially introduced during sequencing, preserving the originality and accuracy of nucleic acid measurements [11]. This capability aids in achieving accurate traceability of nucleic acid quantities, thereby enhancing the consistency and comparability of measurement results.

**Figure 1:**
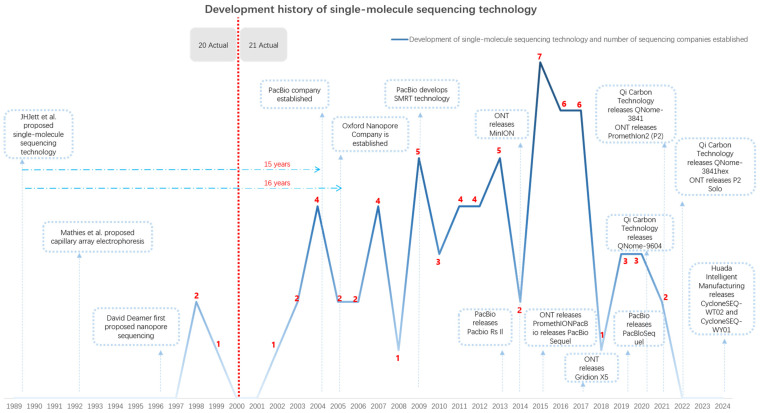
Development of single-molecule sequencing technology.

This can be seen in Figure 1, single-molecule sequencing technology has made remarkable progress since its conceptualization in 1989, pioneering contributions from Pacific Biosciences (PacBio) and Oxford Nanopore Technologies (ONT) offering long-read, real-time sequencing without the need for PCR amplification. PacBio’s SMRT sequencing and ONT’s nanopore-based MinION have transformed nucleic acid analysis by improving accuracy, portability, and throughput, making them invaluable for resolving complex genomes and detecting structural variants. Recent innovations, such as platforms developed by Qi Carbon Technology and Huada Intelligent Manufacturing, emphasize affordability and scalability, further improving measurement consistency and precision. These advancements are critical for nucleic acid metrological traceability, as SMST preserves the originality of nucleic acid measurements, eliminates amplification bias, and ensures accurate quantification—factors essential for achieving reliable comparisons and reproducibility in genomic research and diagnostics.

### 2.1. Modification of Single-Molecule Samples

#### 2.1.1. Library Construction Based on Fluorescence Detection

**Figure 2:**
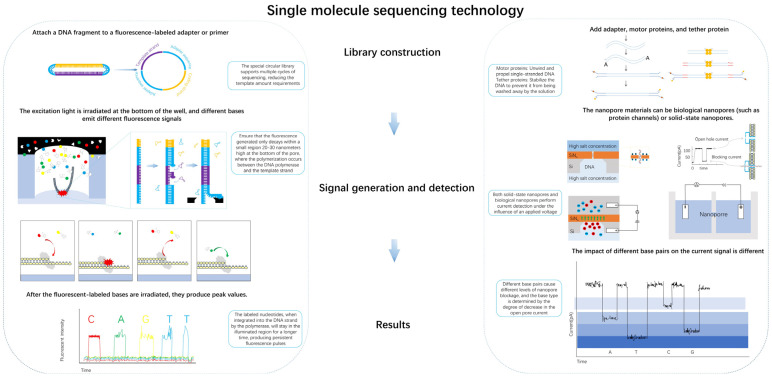
Flow chart of single-molecule sequencing technology. In signal detection, the red, green, yellow, and blue colors represent fluorescently labeled dNTPs, respectively. In library construction, purple represents the template strand, orange represents the coding strand, and blue repre-sents the adapter sequence.

Similar to first- and second-generation sequencing methods, fluorescence signal recognition is also a core technology in single-molecule sequencing. While it also relies on fluorescence signal detection, the underlying technical principles differ, leading to distinct library construction methods. Second-generation sequencing involves bridge PCR amplification, four-color fluorescent reversible terminator synthesis, and laser scanning imaging. This process requires DNA to be fragmented, amplified by bridge PCR to form DNA clusters, and then sequenced. In contrast, single-molecule fluorescence sequencing directly synthesizes DNA strands through the action of polymerase using fluorescently labeled dNTPs, detecting the fluorescence signal in real time to determine the base sequence. As a result, the traditional library construction method, which involves breaking DNA into fragments, is not suitable for single-molecule sequencing. Instead, in single-molecule fluorescence sequencing, the library construction method involves attaching two circular adapters to both ends of double-stranded DNA, thereby forming a circular structure [12,13,14]. During sequencing, DNA polymerase replicates the circular library, allowing each nucleotide in the circular DNA to be detected. The detailed structure of the circular library is shown in Figure 2 above left.

Single-molecule fluorescence sequencing technology utilizes circular libraries primarily to enhance sequencing accuracy and sensitivity. Circular libraries prevent chain breaks and template loss, providing a more stable and sustained fluorescence signal. This in turn increases signal strength, reduces background noise, improves the depth of single-molecule readings, and lowers the error rate. Moreover, circular libraries eliminate template competition, ensuring efficient sequencing of each template. By reading the same molecule in cycles, cyclic libraries can also increase data depth, making them particularly effective for complex or repetitive sequences. However, the construction of circular libraries is relatively complex, requiring specific enzymes or chemical reactions to block DNA molecules. This process is cumbersome and may limit library expansion. Additionally, although the ring structure enhances stability, errors can arise during cyclic reading, particularly in regions with high variability or complex sequences.

#### 2.1.2. Library Construction Based on Electrical Signal Detection

Another key method involves building a library based on electrical signal detection. Since intact nucleic acid molecules cannot pass directly through the nanopore, it is necessary to unwind the double-stranded DNA into a single strand. This is achieved by adding a motor protein, which acts as a propeller, to the nucleic acid molecule, thereby pulling the single-stranded DNA through the nanopore. The DNA is modified by adding a base at the 3′ end, followed by a splice sequence, which is then linked to the motor protein (which facilitates unwinding and propulsion) and a tether protein (which anchors the DNA). When a voltage is applied, the negatively charged single-stranded DNA or RNA molecule is driven through the nanopore, causing a change in current across the membrane. The different bases induce distinct changes in the current, allowing for real-time recording and decoding into a nucleotide sequence.

Depending on the steps and time required to build the library, it can be divided into a standard library [15,16] and transposase library [17]. The transposase method is faster because it consolidates multiple steps, such as DNA fragmentation, end repair, and ligation, into a single reaction, significantly reducing the time required for database construction. One of the main advantages of this method [18] is that it eliminates the need for sample amplification using enzymes, as well as the requirement for fluorescent labeling or other chemical modifications of DNA, thereby simplifying the sample preparation process prior to sequencing. The detailed flowchart for standard library construction and transposase-based library construction is shown in the top right corner of Figure 2 below.

Standard library construction typically involves DNA extraction, fragmentation, end repair, and ligation steps, with the goal of preparing DNA libraries optimized for nanopore sequencing. First, DNA is extracted from the sample, and enzymes are used to fragment the DNA, ensuring the fragments are suitable for nanopore sequencing. The ends of the DNA fragments are then repaired to maintain fragment integrity. Finally, adapters compatible with the nanopore sequencing platform are ligated, enabling efficient identification and reading of the DNA molecules in the nanopore. The main advantage of this standard library construction method is its ease of use and adaptability to various sample types. However, since it is not specifically tailored to the size and structure of DNA fragments, it may impact the sequencing quality in certain cases. An alternative approach is transposase-based library construction, which uses transposase enzymes to directly insert adapters into the DNA fragments. The core of this method is the ability of transposase to introduce random cuts in the DNA and insert the adapters at these sites. Transposase-based libraries generally do not require prior fragmentation or end repair, enabling the direct addition of adapters. A key benefit of this approach is its speed and efficiency, particularly for low-quality or limited DNA samples. Since transposase can rapidly construct libraries, this method is well suited for high-throughput and rapid sequencing. Additionally, transposase-based libraries tend to produce a more uniform fragment size distribution, which improves sequencing accuracy and sensitivity.

### 2.2. Single-Molecule Signal Generation

#### 2.2.1. Zero-Mode Waveguide Hole Based on Fluorescence Signal

**Figure 3:**
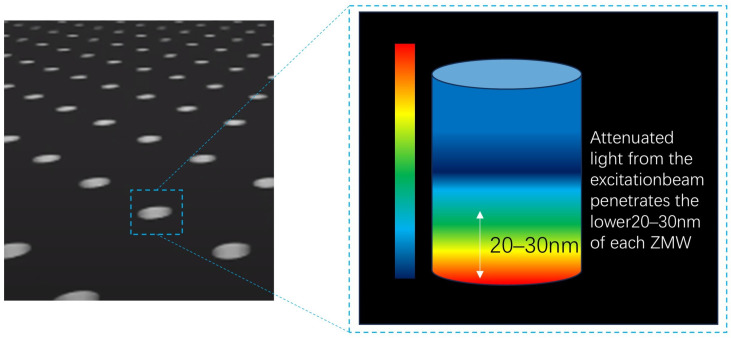
Schematic diagram of the zero-mode waveguide hole structure.

Any linear structure that transmits electromagnetic waves, including light waves, is referred to as a waveguide. When a light wave is introduced into the bottom of a cylindrical waveguide, and the wavelength of the light exceeds 1.7 times the diameter of the waveguide (i.e., the incident wavelength > 1.7 d), the light wave cannot propagate through the waveguide. Instead, it generates an evanescent wave at the waveguide’s entrance. This mode, in which light transmission does not occur within the waveguide, is known as the “zero-mode waveguide”. The schematic diagram of the zero-mode waveguide pore structure is shown in Figure 3 below. A “zero-mode waveguide” (ZMW) is a nanoscale structure that confines light at the bottom of a circular tube [19]. In order to effectively detect fluorescence signals, the appropriate size of a ZMW hole [20,21] with a diameter of about 70 nm and a depth of about 100 nm is selected as the core component of signal generation. This size allows a DNA polymerase molecule to move freely at the bottom of the ZMW without interference from other polymerase molecules. Each ZMW is paired with a single polymerase molecule, and the overall structure consists of a circular hole in an aluminum-clad film mounted on a transparent silica substrate [22]. The ZMW structure also prevents red and green mixed excitation light (532 nm, green; 642 nm, red) from passing through, ensuring that the resulting fluorescence decays only within a small region at the bottom of the hole, approximately 20–30 nm high. This region is where DNA polymerization occurs between the DNA polymerase and the template strand. Biotin molecules on the polymerase bind to streptavidin affinity labels at the bottom of each ZMW hole, anchoring the DNA molecule to the bottom of the hole. Simultaneously, four-color dNTP substrates randomly enter the illuminated region at the base of the ZMW when the laser shines on the transparent bottom plate. As the fluorescently labeled [23] nucleotides diffuse in and out of the illuminated region, they are excited by the laser and emit brief fluorescence peaks. When incorporated into DNA strands by the polymerase, these labeled nucleotides remain in the illuminated region longer, producing prolonged fluorescence pulses. By analyzing these fluorescence pulses and their colors, individual DNA sequences can be identified in a directional manner.

To further enhance the performance of zero-mode waveguide (ZMW) holes in single-molecule fluorescence sequencing, several key issues remain to be addressed. First, the aperture and geometry of the ZMW hole need to be optimized with greater precision to maximize fluorescence signal enhancement while minimizing unnecessary light loss. Second, the selection of waveguide hole material and surface modifications is critical, as surface irregularities or optical losses in the material may lead to signal attenuation or scattering, thereby compromising the accuracy and efficiency of sequencing. To address this, the development of novel optical materials and surface modification techniques will be crucial in reducing noise and improving signal stability. Finally, with advancements in nanofabrication technology, the integration of ZMW holes with high-pass quantization is a promising research direction. This will increase the efficiency and broaden the application of this technology in large-scale genome sequencing.

#### 2.2.2. Nanopore Based on Electrical Signal

Single-molecule sequencing technology based on electrical signal detection analyzes the characteristics of the analyte by measuring the changes in current (such as blocking current amplitude and blocking current duration) when the molecule passes through the nanopore under a certain voltage. A key component of this technology is the nanopore, which can be classified into two types: bionanopores and solid-state nanopores, depending on the materials used and the methods employed for their preparation.

(1) Biological nanopore

**Figure 4:**
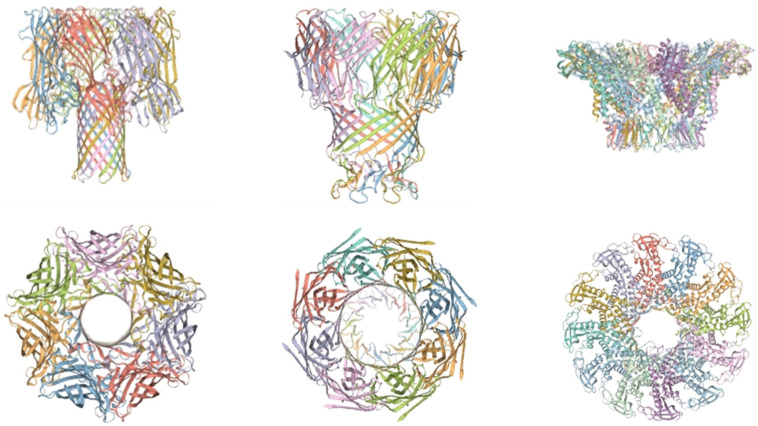
Schematic diagram of the structural models of *α-HL*, *MspA*, and *Phi29* biological nanopores.

Biological nanopores, also known as transmembrane protein channels, are formed through the self-assembly of protein subunits, peptides, or even DNA scaffolds in lipid bilayers or block copolymer membranes. These nanopores exhibit well-defined, highly reproducible sizes and structures, which can be modified using modern molecular biology techniques [24]. The most widely used biological transmembrane channel proteins include Staphylococcus aureus *α-hemolysin (α-HL)*, *Mycobacterium smegmatis toxin protein A (MspA)*, and *phage Phi29*. The schematic diagrams and top views of the above three biological nanopore structures are shown in Figure 4. The *α-hemolysin* channel [25] is approximately 10 nm in length, with a diameter ranging from 1.4 nm to 4.6 nm [26], and was the first nanopore [27] used for biological single-molecule detection. The *MspA* protein [28] efficiently recognizes and translocates single-stranded DNA molecules through nanopores [29,30]. *Phi29* is known for its strong chain-displacement activity and continuous synthesis capability [31].

Compared to solid-state nanopores, biological nanopores are naturally occurring channel proteins that do not require complex chemical synthesis processes. As a result, they offer high flexibility, customizability, and biocompatibility. However, their pore structure is relatively fragile and can be easily affected by environmental conditions, leading to deformation or blockage. This limits their long-term stability and reusability.

(2) Solid state nanopore

In contrast to biological nanopores, which cannot be synthesized naturally, solid-state nanopores, which can be directly manufactured, are becoming increasingly prevalent. Solid-state nanopores are typically created on various carrier materials using preparation methods such as focused ion beam (FIB) etching, transmission electron microscopy (TEM) drilling, chemical etching, and dielectric breakdown [32,33]. Depending on the carrier material, solid-state nanopores can be classified into silicon-based, carbon-based, and two-dimensional nanopores [34]. Taking SiN_x_ as an example, the schematic diagram of the solid-state nanopore structure is shown in the middle of the right half of Figure 2. Silicon nitride (SiN_x_) is the most common material used for silicon-based nanopores. In 2003, researchers first used electron beams to prepare SiN_x_ nanopores on SiN_x_ films [35,36]. Since graphene was first isolated by Geim and Novoselov in 2004 [37], it has garnered extensive attention for its high mechanical strength, large specific surface area, and high electrical conductivity [38]. Boron nitride (BN), a micro–nanostructure material, offers a wider range of physicochemical properties due to its two-element composition [39]. Molybdenum disulfide (MoS_2_) is considered an ideal material for nanopores with the best detection sensitivity and resolution due to its extremely thin structure [40]. Single-layer MoS_2_ exhibits excellent chemical and mechanical stability. Tungsten disulfide (WS_2_) is a type of transition metal dichalcogenide (TMD). Sub-nanometer-sized holes can be created in monolayer WS_2_ films through electron beam irradiation. These holes are stabilized by W-W bonding and bond rotation, indicating that WS_2_ films have the potential to be used for fabricating nanopores with precisely controlled sizes.

Compared to biological nanopores, solid-state nanopores offer advantages such as superior mechanical properties, strong stability, and ease of shape control [41,42]. The size and shape of these nanopores can be precisely adjusted at the sub-nanometer level, enabling accurate control of pore apertures. However, solid-state nanopores tend to produce higher noise due to material properties, and the rapid translocation of DNA molecules through the pores can result in lower signal recognition rates.

### 2.3. Single-Molecule Signal Detection

#### 2.3.1. Fluorescence Signal Detection

One of the core components of the single-molecule fluorescence signal detection system is the detection chip, which contains between 150,000 and 8 million zero-mode waveguide (ZMW) holes [43]. During sequencing, the library to be sequenced is added to the chip, where it randomly enters the sequencing unit of the ZMW. The probability of a library molecule entering a small hole follows the Poisson distribution principle. A single polymerase enzyme is fixed to the glass plate at the bottom of each ZMW. Four different fluorescently labeled dNTP substrates are then introduced into the ZMW pore. Each nucleotide base is tagged with a corresponding fluorescent dye molecule, which attaches to the phosphate group at the nucleotide’s 5′ end.

During the synthesis process, when a free dNTP molecule approaches the bottom of the ZMW, it is captured by the polymerase fixed at the bottom. If the dNTP is successfully paired with the template chain, it will be illuminated by excited light and emit fluorescence, which will be recorded by the signal collector. After the synthesis is completed, the fluorophores will naturally fall off along with the phosphate groups, and the cut fluorescent dye molecules will spread out the detection volume. The fluorescence signal is no longer detected, and sequencing is completed through a continuous cycle of this process [44,45,46]. When the dNTPs with different fluorescent labels are incorporated into the template strand in each ZMW, the fluorescence emitted at different wavelengths can be excited. The type of dNTP added is determined based on the wavelength and peak intensity of the emitted fluorescence. If the base is modified, the polymerase’s progression is slowed, and the distance between adjacent fluorescence peaks increases. This allows for the direct detection of methylation and other modifications [47,48]. There are two sequencing modes for single-molecule fluorescence detection: Highly Accurate Long Reads (HiFi reads) [49] and Continuous Long Read (CLR) [50]. HiFi reads adopt a model of circular consensus sequencing (CCS); that is, consistent sequences can be obtained from multiple passes of a single template molecule, and precise reads can be produced from individual noisy subreads. CCS mainly applies DNA insertion shorter than 2 kb. In contrast, the CLR sequencing model enables high-quality assembly of complex genomes. CLR focuses on achieving longer read lengths but with lower accuracy compared to CCS. As a result, CLR is better suited for research requiring long read lengths but with less stringent accuracy requirements, such as genomic structural variation detection or de novo genome assembly.

#### 2.3.2. Electrical Signal Detection

The concept of single-molecule detection using nanopore systems was independently proposed in the 1980s by several research groups, including those led by David Deamer, George Church, and Hagan Bayley [51,52]. The underlying hypothesis was based on the theory that oligomers, when exposed to an electric current, could pass through protein nanopore channels. As they move through the pore, the characteristics of their base composition would disrupt the current, allowing for base sequence identification. Electrical signal detection in nanopore systems involves placing the nanopore in two electrolyte-filled chambers (such as a KCl or Ag/AgCl system) [53,54]. When an applied voltage is used, a single-stranded nucleotide sequence passes through the nanopore, and the base molecules impede ion flow. This causes characteristic changes in the current signal. Since different bases affect ion flow in distinct ways, the fluctuations in the current can be analyzed to identify the bases passing through the nanopore, thereby allowing for base sequence reading. Because the current fluctuations caused by the movement of base molecules are weak—on the order of picoamperes [55]—the electrical signal generated by the nanopore is typically detected using I-V conversion [56,57]. The core principle of I-V conversion involves a transresistance amplifier. According to the “virtual short” and “virtual break” characteristics of the ideal model of the operational amplifier, the output voltage can be approximated as

(1) $$V_{0}=I_{f}\times R_{f}=I_{i}\times A_{f}=-I_{i}\times R_{f}$$

In Formula (1), *V*_0_—output voltage, *I_f_*—feedback current, *R_f_*—feedback resistance, *I_i_*—input current to be measured, and *A_f_*—amplification factor. Then, the current to be measured Ii can be expressed as

(2) $$I_{i}=-\frac{V_{0}}{R_{f}}$$

According to Formula (2), the amplification factor of the input current to be measured by the I-V transformation method is determined by the resistance value of the feedback resistance.

The characteristic of this conversion method is that the response time of the signal is short, the change of the input signal can be quickly detected, and the result can be converted to the output end, so it is suitable for the characteristics of fast change of the nanopore signal.

### 2.4. Single-Molecule Sequencing Data Analysis

Although the signal generation and detection methods of single-molecule sequencing technologies differ significantly, the fundamental logic of data analysis remains similar. Whether the signal is generated through electrical detection (FAST 5 format) [58] or fluorescence detection (HDF 5 format) [59], the data are ultimately converted into a base sequence through base calling using machine learning algorithms (such as the HMM model) or neural network algorithms (e.g., the RNN model). The result is typically a standard FASTQ file format for subsequent analysis.

The basic workflow of single-molecule sequencing data analysis closely mirrors that of second-generation sequencing, involving data quality control, filtering, and comparison. However, single-molecule sequencing faces the challenge of a high error rate, especially when obtaining ultra-long reads. Some studies have reported that the global sequencing error rate of certain single-molecule platforms is around 11%, primarily due to base insertions and deletions. To reduce this error rate, various base recognition models have been developed to improve accuracy. For example, the high accuracy (HAC) model combines convolutional neural networks (CNN) with long short-term memory (LSTM) networks, achieving an average base quality of Q20 (99.0%). The Super Accuracy (SUP) model further enhances accuracy by incorporating deeper networks, such as bidirectional LSTM or Transformer models, yielding an average base quality of Q23 (99.5%) [60]. To improve the reliability of sequence alignment for detecting complex structural variations, Professor Michael Schatz’s team, in collaboration with Pacific Biosciences, developed specialized sequence alignment tools for single-molecule sequencing data using seed-extension algorithms [61,62]. Additionally, Schatz’s team at Cold Spring Harbor Laboratory created a hybrid error correction method that combines long-read data from third-generation sequencing with short-read, high-precision data from second-generation sequencing to correct errors in long-read sequences [63]. Thus, through advancements in bioinformatics, including algorithm and model improvements, the accuracy of sequence information can be significantly enhanced.

### 2.5. Performance Comparison of Single-Molecule Sequencing Methods

Single-molecule fluorescence sequencing and single-molecule nanopore sequencing are two primary methods for genome sequencing at the single-molecule level. Single-molecule fluorescence sequencing involves labeling the DNA with a fluorescent signal and using real-time fluorescence detection to infer the nucleotide sequence. Its advantage lies in the ability to rapidly read long sequences with a low error rate. However, its main limitation is the reliance on complex chemical labeling and fluorescence detection systems, making the equipment expensive. In contrast, single-molecule nanopore sequencing directly reads gene sequences by monitoring current changes as DNA molecules pass through a nanopore, without the need for chemical markers, which simplifies the sequencing process. The primary drawback of this technology is that decoding the current signal requires complex algorithms, and due to limitations in pore size and material, it is susceptible to interference from environmental factors, leading to relatively low sequencing accuracy.

A detailed performance comparison of the two sequencing methods is shown in Table 1.

## 3. Advantages and Applications of Single-Molecule Sequencing Technology

### 3.1. Advanced Nature

Single-molecule sequencing technology enables direct reading of nucleotide sequences at the single-molecule level. Compared to first- and second-generation sequencing technologies, single-molecule sequencing offers several significant advantages: (1) a very long read length: Single-molecule sequencing technology can produce a read length far exceeding that of traditional technology [73,74], which is crucial for reducing the number of Contig after sequencing and reducing the subsequent genome splicing and annotation workload. (2) No need for PCR amplification: Single-molecule sequencing directly sequences individual DNA molecules, avoiding amplification errors and GC bias [75,76]. This reduces experimental errors and improves data quality. (3) Direct detection of RNA: Single-molecule sequencing technology can directly sequence RNA molecules without the need for reverse transcriptional steps [77,78], which provides a new, more accurate, and comprehensive tool for RNA biology research. (4) Real-time sequencing: Single-molecule sequencing technology supports real-time data detection and analysis [79,80], which means that data can be analyzed in real time during the sequencing process without waiting for the entire sequencing to end. Based on these advantages, single-molecule sequencing not only provides more reliable nucleic acid metrology data and improves accuracy but also ensures that nucleic acid regions with extreme base compositions are unaffected by palindromic sequences. This lays a foundation for simplifying the nucleic acid metrology process and provides more accurate information.

### 3.2. Applications

Based on the above advantages of single-molecule sequencing, it has shown significant advantages and wide application prospects in genome sequencing, transcriptome sequencing, epigenetics, and structural variation detection, providing important data and a theoretical basis for biological analysis and clinical research.

In genome sequencing, third-generation sequencing technology has the advantage of an ultra-long reading length, which can reduce the difficulty of stitching complex sequences and obtain longer genome sequence fragments. Liang Xili et al. [81] took the Zhejiang strain of Nosema Bombyx mori as the research object and extracted a high-quality Nosema genome for third-generation sequencing to obtain more complete genome information of Nosema bombyx mori. In terms of the transcriptome, third-generation sequencing makes it possible to infer the structure of full-length transcripts without the need for transcription reconstruction. Halstead et al. [82] conducted a three-generation sequencing analysis of 32 tissues of adult male and female Hayford cattle and found that their testicles exhibited the most complex transcriptional profiles of all the sequenced tissues, which showed that three-generation sequencing could provide a more detailed annotation of the transcriptome of cattle. Based on the long-read characteristics, Gao et al. [83] combined SMRT with Illumina sequencing to study the transcriptome of Eimeria and obtained many long-read isomers, from which a series of lncRNAs, AS events, APA events, and fusion transcripts were identified. It helps to understand the pathogenesis of pathogens and potential drug and vaccine targets. In epigenetic detection, single-molecule sequencing technology can directly detect epigenetic information such as DNA methylation modification, which is of great significance for understanding the regulatory mechanism of gene expression and epigenetic changes in the occurrence and development of diseases. Choy LYL et al. [84] used single-molecule sequencing to sequence the plasma DNA of cancer patients and performed fragment size and direct methylation analysis for each molecule, developed and analyzed indicators that could reflect the single-molecule methylation patterns associated with cancer, and discovered cfDNA, which provided the possibility for direct methylation analysis of cancer liquid biopsy. Ohshiro et al. [85] determined the methylation sites and rates of 5mC and m6A in hsa-miR-200c-5p extracted from colorectal cancer cells by single-cell sequencing technology. The use of long-read technology can more accurately detect tumor genomic variation, including structural variation, copy number change, gene fusion, and so on. For example, Martignano et al. [86] used low-coverage nanopore sequencing to detect the copy number changes of plasma nucleic acid molecules from cancer patients and completed genome-wide molecular karyotype detection of six lung cancer patients and four healthy subjects with only 2 million readings and found common copy number changes associated with lung cancer.

## 4. Challenges of Single-Molecule Sequencing Technology and Significance of Metrological Traceability

### 4.1. Single-Molecule Sequencing Challenges

Single-molecule sequencing technology has brought breakthrough advantages, this technology has attracted wide attention for its single-molecule detection, long reading length, real-time analysis, etc., but in the process of fully realizing its potential, there are still some challenges. (1) Noise interference: The signal generated by single-molecule detection is relatively weak, and the noise in the process of signal recognition will seriously affect the effectiveness of the sequencing results. Taking electrical signal detection as an example, preamplifier noise [87] is the main noise source of the detection system. At the same time, there is also electrode noise [88] (internal noise generated by electronic, electrical, and mechanical equipment, as well as external noise resulting from capacitive coupling, electromagnetic coupling, and current coupling) and nanopore noise [89,90] (surface charge fluctuations, instability of ion flow in the electrolyte, and device capacitance [91]). In particular, changes in the surface charge density affect the strength of the current flowing through the nanopore. If the noise levels are too high, base information may be obscured by the noisy signal, resulting in missing sequencing data. (2) Chemical reaction bias: In fluorescence detection technology, incorrect synthesis of polymerase will lead to DNA strand synthesis errors [92], and uneven incorporation of fluorescently labeled nucleotides will also lead to inaccurate signal recording and affect sequencing quality. These errors are mainly manifested as insertion and deletion. (3) Low resolution: In terms of spatial and temporal resolution, it is still difficult to accurately identify a single base. In particular, the increase in homopolymers and tandem repeat regions [93], which are characterized by single-base repeats on the genome and tandem repeats with relatively constant short sequences as repetition units, will introduce indel errors and reduce sequencing accuracy. For example, for a sequence of eight repeating units, the error rate within the homopolymer region (5.6%) was higher than in its adjacent region (3.6%). When the repeating unit was extended to 76, the error rate within the homopolymer region increased to 10 percent, which was 78 percent higher than in adjacent regions.

Currently, the efficient and reliable preparation of single-molecule sequencing remains a significant hurdle. To address this, advancements in precise processing technology, optimization of polymerase activity, and the realization of atomic-level control for high reproducibility and stable synthesis are essential for transforming the scientific potential of single-molecule sequencing into practical applications.

### 4.2. Measurement Traceability

Nucleic acid metrological traceability is a cornerstone for ensuring accuracy. As biotechnology continues to advance rapidly, the importance of nucleic acid metrological traceability is becoming increasingly prominent. The advent of single-molecule sequencing technology has made it possible to directly measure nucleic acid sequences. The significance of this technology for nucleic acid measurement and traceability is reflected in the following two key aspects: (1) Traditional sequencing methods rely on PCR amplification, which can introduce errors such as base mismatches or preferential amplification of certain base sequences. These errors are often amplified during the PCR process, leading to over-amplification or under-amplification of specific regions. Single-molecule sequencing technology enables comprehensive coverage of various species or variants within the entire nucleic acid sample by sequencing each molecule individually. This is crucial for high-precision nucleic acid metrology and sample traceability, effectively reducing traceability transfer and improving the accuracy of metrological traceability. (2) Single-molecule sequencing technology allows for the direct sequencing of individual bases, offering a new approach to nucleic acid metrology. Traditional methods of nucleic acid measurement typically involve quantifying DNA copy numbers through labeling techniques, which provide indirect measurements. In contrast, single-molecule sequencing directly measures base characteristics, providing a more precise and detailed understanding of nucleic acid sequences. This opens up a new dimension of traceability in nucleic acid sequence measurement, enabling the construction of a more reliable and accurate nucleic acid measurement traceability system.

## 5. Summary and Outlook

As a groundbreaking method of gene sequencing, single-molecule sequencing technology has garnered significant attention in the academic community. Current research worldwide is focused on improving the performance, accuracy, and throughput of sequencing devices. However, relatively few studies have explored the relationship between single-molecule sequencing technology and nucleic acid sequence metrology. This paper provides an in-depth discussion of the theoretical foundations, technical approaches, and unique advantages of single-molecule sequencing technology while also analyzing and exploring its interaction with nucleic acid metrology. As a representative of third-generation sequencing technology, single-molecule sequencing holds broad market potential and application prospects, particularly in the fields of life sciences and medical research, due to its unique advantages. Looking ahead, single-molecule sequencing technology is expected to evolve in several key areas: higher accuracy, ultra-high precision detection of single molecules, longer throughput, greater reliability of long fragment sequencing, more accurate signal recognition, and deeper integration with artificial intelligence (AI) and big data analysis. These advancements will bring revolutionary changes to the field of nucleic acid metrology. This technology enables the direct reading of nucleotide sequences, providing direct observation of DNA and RNA molecules, thereby enhancing the accuracy and reliability of nucleic acid measurements and establishing a new system of sequence measurement and quantitative value traceability.

The development of single-molecule sequencing technology not only offers new possibilities for nucleic acid metrology but also significantly advances progress in life sciences and medical research. In turn, the ongoing development of nucleic acid metrology will create broader applications for single-molecule sequencing technology. The mutual advancement of both fields signals that they will play an increasingly crucial role in each other’s development. With continued technological progress, single-molecule sequencing is expected to hold greater application value in the collaborative evolution of nucleic acid metrology.

## Figures and Tables

**Table 1:** Single-molecule sequencing performance comparison table.

Peculiarity	Biological/Solid-State Nanopore		Zero Mode Waveguide Hole
Material selection	*α-HL*, *MspA*, *Phi29*	Silicon based nanopore, carbon material nanopore, BN, MoS_2_, WS_2_	SiO_2_, Si, polymer materials
Material characteristics	High flexibility, customizability, biocompatibility	Good thermal stability, good chemical stability, controllable pore size, many kinds of optional materials	Highly localized light field, low loss transmission, surface fluorescence enhancement effect
Aperture thickness	1.2 nm–3.6 nm	0.34 nm–3 nm	50 nm–250 nm
Build koot point	Add motor proteins and end bases		A ring library is formed by adding ring joints
Sequencing process	Library construction—signal detection—electrical signal collection—sequence analysis		Library construction—signal detection—fluorescence number collection—fluorescence signal conversion—sequence analysis
Principle of sequencing	Electrical signal based		Fluorescent signal-based
Reads	The current maximum is 2 Mb; using standard DNA up to 4 Mb (4 million bases)		Maximum read length: more than 50 kb; single run output: more than 100 Gb
Average reads	23 kb–100 kb		8 kb–15 kb
Flux	1 Gb–150 Gb		500 M–100 Gb
Accuracy	99.75%		99.99%
Sequencing quality	Q26		Q30
Error type	Insertion, deletion, substitution		Insertion, deletion
Data generation rate	fast		quicker
Direct RNA sequencing	Direct RNA sequencing without reverse transcription		Reverse transcription is required to convert the RNA into cDNA, which is then sequenced
Real-time analysis	Support real-time data analysis workflow, save overall time, more effective for time-sensitive experiments, e.g., pathogen detection, tumor resistance screening, SV site analysis		Preliminary analysis can be performed on the run, including deep learning algorithms to measure DNA methylation status, bar code resolution, and conversion to standard BAM format, without waiting for the entire sequencing process to finish
Cost	USD 0.01/1 bp–USD 0.1/1 bp		USD 0.05/1 bp–USD 0.2/1 bp
Equipment volume	small		big
Ease of operation	easy		complexity
Representative product	QNome-3841, QNome-3841hex, QPursue-6k, QPursue-6khex PromethlON, GridlON, MinlON CycloneSEQ-WT02, CycloneSEQ-WY01		1. PacBio: PacBio RS llP6-C4, PacBio Sequel, PacBio Sequel ll 2. Helicos BioScience: HeliScope GensKey S, GensKey M
Reference	[64,65,66,67,68]		[69,70,71,72]
